# Loneliness, insomnia symptoms, social jetlag, and vitamin D deficiency in relation to mental health problems in Japanese female university students: a cross-sectional study

**DOI:** 10.1186/s40101-025-00403-9

**Published:** 2025-07-09

**Authors:** Nodoka Yamashita, Shioka Ishii, Yoriko Kotoku, Takuya Shuo, Hiromi Eto, Hideaki Kondo

**Affiliations:** 1 Graduate School of Biomedical Sciences, Nagasaki University, 1-7-1 Sakamoto, Nagasaki-City, 852-8520 Japan; 2 Department of Reproductive Health, Institute of Biomedical Sciences, Nagasaki University, 1-7-1 Sakamoto, Nagasaki-City, 852-8520 Japan; 3 Faculty of Health and Medical Sciences, Hokuriku University, 1-1 Taiyogaoka, Kanazawa, Ishikawa, 920-1180 Japan; 4 Department General Medicine, Nagasaki University Hospital, 1-7-1 Sakamoto, Nagasaki-City, Nagasaki, 852-8501 Japan

**Keywords:** Insomnia, Loneliness, Mental health, 25-hydroxyvitamin D, Social jetlag, Vitamin D

## Abstract

**Background:**

Vitamin D deficiency is highly prevalent among Japanese female university students. Vitamin D deficiency is associated with physical and mental health problems, including sleep disorders. This study aimed to clarify the relationship between vitamin D deficiency and sleep and mental health problems among Japanese female university students.

**Methods:**

Participants were 224 female university students. Blood levels of 25-hydroxyvitamin D [25(OH)D] were measured using liquid chromatography-tandem mass spectrometry for vitamin D assessment. Mental health was assessed using the K6. Sleep–wake status as a factor related to mental health was assessed using the Athens Insomnia Scale (AIS) and Munich ChronoType Questionnaire. Loneliness was assessed using the Three-Item Loneliness Scale. Factors predicting mental health problems with a K6 score ≥ 5 were explored using the Mann–Whitney U test, Fisher’s exact probability test, and classification and regression tree (CART) analysis.

**Results:**

The median (interquartile range) serum 25(OH)D concentration was 14.5 (11.8–18.3) ng/mL. Of the participants, 80.8% had vitamin D deficiency (25(OH)D < 20 ng/mL), and 26.3% had severe vitamin D deficiency (25(OH)D < 12 ng/mL). In total, 41.1% had mental health problems with a K6 score of ≥ 5. Although there was no significant association between vitamin D deficiency and sleep–wake problems, vitamin D deficiency was more prevalent among those with K6 scores ≥ 5 (*P* = 0.02). Compared to those with K6 < 5, those with K6 ≥ 5 had significantly higher Loneliness and AIS scores (*P* < 0.001), greater social jetlag (*P* = 0.03), shorter sleep duration on weekdays (*P* = 0.03), and lower serum 25(OH)D concentration (*P* = 0.02). In the CART analysis, the algorithm was set in the order of Loneliness score ≥ 6, AIS score ≥ 7, social jetlag ≥ 150 min, and serum 25(OH)D concentration < 14 ng/mL, and the target accuracy (95% confidence interval: CI) was 76.5 (70.3–81.9)%, and sensitivity and specificity (95% CI) were 62.2 (51.4–72.2)% and 86.3 (79.2–91.6)%, respectively.

**Conclusions:**

Loneliness, insomnia symptoms, social jetlag, and vitamin D deficiency were associated with mental health problems among Japanese female university students.

**Supplementary Information:**

The online version contains supplementary material available at 10.1186/s40101-025-00403-9.

## Background

Biological vitamin D is classified as vitamin D_3_, which is synthesized in the skin (some is derived from animal food sources), or vitamin D_2_, derived from plant food sources [1]. Both are hydroxylated at the 25-position in the liver to 25-hydroxyvitamin D [25(OH)D] and hydroxylated at the 1-position in the kidney to biologically active 1, 25-hydroxyvitamin D [1, 25(OH)2D]0.1, 25(OH)_2_D is rapidly synthesized by enzyme induction under vitamin D deficiency, resulting in fluctuating blood levels. 25(OH)D, which has a half-life of 2–3 weeks [2, 3], is an indicator of vitamin D deficiency. Although the cutoff values for vitamin D deficiency vary among countries and organizations, 25(OH)D < 20 ng/mL is considered a state of mild deficiency, and < 10–12 ng/mL is often considered a state of severe deficiency [4, 5].

Vitamin D deficiency is highly prevalent: a meta-analysis based on 308 epidemiological studies including a total of 7.95 million people in 81 countries published between 2000 and 2022 showed that the prevalence (95% confidence interval: CI) of vitamin D deficiency was 44.7 (44.7–50.8)%, and 60.2 (55.5–64.9)% in the 20–40°N region, including Japan, with similar rates in each age group [6]. In 2019, vitamin D deficiency was more frequently reported (79%) in one-year health checkups (*n* = 5,481) [7]. In our survey of 203 pregnant women, 79.8% were also deficient [8]. Vitamin D deficiency has been extensively studied as a public health problem across all age groups.

The association between vitamin D deficiency and physical and mental disorders is well established. Vitamin D deficiency increases the risk of obesity [9, 10], metabolic syndrome [11, 12], type 2 diabetes [13, 14], atrial fibrillation [15], cardiovascular disease [16], and breast and thyroid cancer [17, 18]. Additionally, in patients with type 2 diabetes, vitamin D deficiency or insufficiency increases the risk of all-cause mortality and death from cardiovascular disease [19]. It is also associated with dementia [20] and depression [21–23]; severe vitamin D deficiency is associated with resistance to the treatment of psychiatric symptoms [21]. Many vitamin D replacement therapies have been implemented for various diseases and conditions. Although they do not necessarily contribute to improvements in all outcomes, their efficacy and improvement have been reported in many cases [5, 24, 25].

A considerable body of knowledge has accumulated on the pathophysiology of central nervous system disorders in relation to vitamin D [26–28]. A vitamin D metabolic pathway exists in the central nervous system, where active vitamin D is synthesized and inactivated. Vitamin D receptors are widely expressed in the brain. These receptors are crucial for regulating numerous gene expressions and are integral to the development and functioning of the nervous system. In addition, vitamin D plays a role in modulating immune function, managing inflammation, and affecting central nervous system diseases. Although vitamin D alone does not account for all central nervous system-related issues, it is a critical component in the pathophysiology of various diseases.

Vitamin D deficiency is particularly prevalent among Japanese female university students; however, the association between vitamin D deficiency and mental health in this population has not been clarified. Vitamin D deficiency (25 (OH) D < 20 ng/mL) was found in 10–15% of female university students in the United States [29], but was significantly higher in Japanese female university students (57–77%) [30, 31]. In the U.S. study, serum vitamin D levels were higher in Caucasians and non-Hispanics than in other races [29] and were influenced by differences in skin melanin pigment levels [32]. Although vitamin D deficiency or insufficiency has been shown to be associated with depressive symptoms regardless of race, Caucasians accounted for more than 80% of the participants in that study, with Asians accounting for approximately 10% of the sample [29]. Therefore, the relationship between vitamin D deficiency and mental health among Japanese female university students needs to be further clarified.

The effects of vitamin D deficiency on sleep and wakefulness need to be clarified.

Vitamin D deficiency or insufficiency is associated with sleep–wake problems, such as short sleep duration, poor sleep quality, insomnia symptoms, and obstructive sleep apnea [33]. Vitamin D deficiency is also associated with restless legs syndrome (RLS) [8, 34]. While the potential association between low blood vitamin D levels and an evening chronotype have been reported [35], this association requires further verification. Additionally, our literature search did not clarify its relationship with social jetlag. Moreover, no studies investigating vitamin D deficiency and sleep–wake problems in female university students were found. Therefore, the relationship between vitamin D deficiency and insomnia and other sleep–wake problems in this age group also requires clarification.

This study aimed to clarify the prevalence of vitamin D deficiency among Japanese female university students and explore the association between vitamin D deficiency and mental/sleep health. In the widely used ligand-binding method for 25(OH)D blood concentration measurement, nearly 10% of subjects may be below the measurement sensitivity [8]. Therefore, liquid chromatography-tandem mass spectrometry (LC–MS/MS) was used to measure blood 25 (OH) D levels, which is capable of accurately measuring even lower levels. Data collection was conducted from 2021 to 2023; therefore, the impact of the COVID-19 pandemic could not be avoided. A meta-analysis found that loneliness increased during the COVID-19 pandemic [36]. A meta-analysis of studies on medical students found that loneliness was associated with mental health problems [37]. It has also been shown that loneliness is related to sleep problems [38]. In the present study, we also investigated loneliness to consider the relationship between loneliness and mental/sleep health. Although no clear relationship between vitamin D deficiency and sleep–wake problems was found, mental health problems were associated with loneliness, insomnia symptoms, and social jetlag, in addition to vitamin D deficiency.

## Methods

### Participants, study design, and ethical considerations

In this study, 281 nursing undergraduates from two universities in Nagasaki Prefecture were invited to participate between 2021 and 2023. Students receiving active vitamin D treatment, those treated for sleep–wake disorders, and those who were pregnant or had a history of pregnancy were excluded. The participants were provided written and oral explanations of the study and consented to participate. A total of 224 (79.7%) participants were included in the analysis after excluding 13 who withdrew consent, 2 who could not have their blood samples taken, and 14 who could not be contacted; initially, consent was obtained from 253 (90.0%) participants. Participants were asked to respond to a questionnaire regarding background information, mental health, and sleep. Blood samples were collected. This study was conducted in accordance with the Declaration of Helsinki and the Ethical Guidelines for Medical Research Involving Human Subjects. It was reviewed and approved by the Ethics Committee of the Nagasaki University Graduate School of Biomedical Sciences (approval no. 21111102–4).

## Measures

### Sociodemographic information

The following background information was obtained: age, living arrangements, dietary intake, drinking habits, smoking habits, outdoor activity time, exercise habits, parasol and sunscreen use, vitamin supplement intake, menstrual status, menstrual cycle, and premenstrual syndrome.

### Munich Chrono Type Questionnaire

The Japanese version of the Munich ChronoType Questionnaire confirms sleep habits on weekdays and weekends, calculates the mid-sleep time (MST) to estimate chronotype, and calculates social jetlag (SJL) [39–41]. Weekday sleep duration (weekday SD) and weekend sleep duration (weekend SD) were calculated from the time of falling asleep and the time of last awakening, respectively. The average weekly sleep duration (average SD) was calculated from the number of weekdays and weekends. SJL was calculated from the MST. The MST, the midpoint between the time of falling asleep and the time of final awakening, was calculated for weekdays and weekends. The relative SJL and its absolute value were calculated by subtracting the weekday MST from the weekend MST. Weekend MST was used to assess the chronotype of each individual. MSTs of the participants were divided into three groups such that the number of MSTs was approximately equal, and the early group was categorized as morning, intermediate, or evening. However, when the sleep duration on weekends was longer than the average SD, the weekends MSTs was corrected as follows: corrected weekend MST = weekend MST—(weekend SD—average SD)/2.

### Athens Insomnia Scale

The Japanese version of the Athens Insomnia Scale (AIS) is an 8-item self-administered questionnaire that measures insomnia severity [42, 43]. The questionnaire asks for responses to insomnia symptoms and sleep-related daytime problems during the previous month. Each item is scored from 0 to 3, and the total score ranges from 0 to 24, with higher scores indicating greater insomnia severity. The cut-off point between normal subjects and insomnia is 6 points. The severity of insomnia is determined as follows: 6–9 points mild, 10–15 points moderate, and 16–24 points severe [44]. In the present study, participants with a total score of six or more points were considered to have suspected insomnia.

### Cambridge-Hopkins questionnaire short form 13

The Cambridge-Hopkins questionnaire short form 13 is a self-administered questionnaire to screen for RLS [45, 46]. It consists of 10 items for RLS screening and 3 items to check the severity, frequency of occurrence, and age of onset. According to the scoring manual, RLS is classified into three categories: definite RLS, probable RLS, and non-RLS. In the present study, definite and probable RLS were defined as RLS.

### K6

The Japanese version of the K6 is a 6-item questionnaire that assesses mental health [47, 48]. Each item is scored from 0 to 4, and the total combined score ranges from 0 to 24, with higher scores indicating more problems. The cut-off value for screening individuals with serious mental illnesses is 12/13 [49]. The sensitivity and specificity for mood and anxiety disorders and suicidal behavior in the DMS-IV have been reported to be 0.75–1.00 and 0.79–0.80, respectively, by setting the cutoff value to 4/5 [50]. In the present study, a K6 score ≥ 5 was defined as having mental health problems, and ≥ 13 as having serious mental health problems.

### Three-Item Loneliness Scale

The Japanese version of the Three-Item Loneliness Scale is a 3-item self-administered questionnaire that measures loneliness [51, 52]. The survey was designed to be easily completed, assuming a large-scale survey using telephones or other means. Each item is scored from 1 to 3, and the total score ranges from 3 to 9, with higher scores indicating a stronger sense of loneliness.

### Blood sampling and analysis

Blood was collected in 9-and 4-mL tubes for serum separation. The serum was centrifuged and divided into two tubes. One was frozen at −20 °C and collected by SRL, an external laboratory, on the day of blood collection and transported to the laboratory. The other bottle of serum was stored at −80 °C and transported to Hokuriku University for 25(OH)D measurement using LC–MS/MS. Total protein, albumin, Cr, Ca, P, Fe, ferritin, and intact parathyroid hormone (PTH) levels were measured by the external laboratory. Serum intact PTH levels were measured using an electrochemiluminescence immunoassay. When the serum albumin concentration was less than 4 g/dL, the calcium levels were corrected for albumin. Serum ferritin levels were measured to assess the influence of iron deficiency on RLS. Intact PTH was also measured because high vitamin D deficiency tends to cause secondary elevation of PTH levels, even when blood calcium levels are normal.

Measurement of 25(OH)D using LC–MS/MS was performed at the Hokuriku University Instrumental Analysis Facility. Samples were deproteinized in methanol/zinc sulfate as preprocessing before solid-phase extraction was performed using an Oasis PRiME HLB (Waters Corp., MA, US). The LC–MS/MS system used an LCMS-8045 triple-quadrupole mass spectrometer coupled with a Nexera X2 high-performance liquid chromatography system (SHIMADZU Corp, Kyoto, Japan); 25(OH)D_2_ and 25(OH)D_3_ were separated on a reverse-phase column (Shim-pack Velox SP-C18, SHIMADZU Corp, Kyoto, Japan) and underwent electrospray ionization before the assay was performed using multiple reaction monitoring. The 25(OH)D_2_ and 25(OH)D_3_ calibrators and deuterated internal standards used were CertiMass Reference Standards (IsoSciences LLC, Ambler, PA, US). Quality control for 25(OH)D_2_ and 25(OH)D_3_ serum level measurements were performed using the ClinCal Serum Calibrator Set (RECIPE, München, Germany). The %CV of this measurement system was 3.2%, the mean % bias was 2.8%, and 83.3% of the measurements had a mean % bias <|5%|, indicating adequate assay performance in a routine laboratory. These values satisfy the reference laboratory criterion of a CV of ≤ 5% and have an accuracy similar to the standard mean % bias criterion of ≤|± 1.7%| [53, 54].

25(OH)D_2_ and 25(OH)D_3_ levels were measured, and 25 (OH) D was used as the sum of the two. Serum 25(OH)D concentration was divided into three groups: severe deficiency (< 12 ng/mL), mild deficiency (12–20 ng/mL), and ≥ 20 ng/mL. A previous study of pregnant women in Nagasaki Prefecture confirmed that serum 25(OH)D concentrations were significantly lower from November to spring [8], and serum 25(OH)D concentrations were compared in four groups from January to March, April to June, July to September, and October to December.

### Statistics

The data were analyzed using EZR version 1.68 [55] based on R version. 4.3.1 (https://www.r-project.org/) and modified R Commander version 4.4.2 (https://home.hirosaki-u.ac.jp/pteiki/r/) based on R ver. 4.2.2. Normality was evaluated using the Shapiro–Wilk test. Continuous variables for which normality was confirmed are expressed as mean (standard deviation), and continuous variables for which normality was not observed are expressed as median (interquartile range [IQR]). For comparisons between two groups of continuous variables, an uncorrelated t-test was used for normally distributed variables, and the Mann–Whitney U test was used for non-normally distributed variables. For comparisons between three or more groups of continuous variables, a one-way analysis of variance (ANOVA) was used for variables that showed normality, the Kruskal–Wallis test was used for variables that showed non-normality, and the Bonferroni method was used for multiple comparisons. Fisher’s exact probability test was used to test the independence of the nominal variables. The significance level was set at *P* < 0.05.

Classification and regression tree (CART) analysis [56] was used to find the cutoff values of each parameter related to mental health problems with K6 ≥ 5 in an exploratory manner. Variables that were significant in a comparison between two groups, K6 ≥ 5 and K6 < 5, were used as input variables. The accuracy, sensitivity, and specificity of the employed algorithms and their 95% confidence intervals (CIs) were also calculated.

## Results

**Fig. 1:**
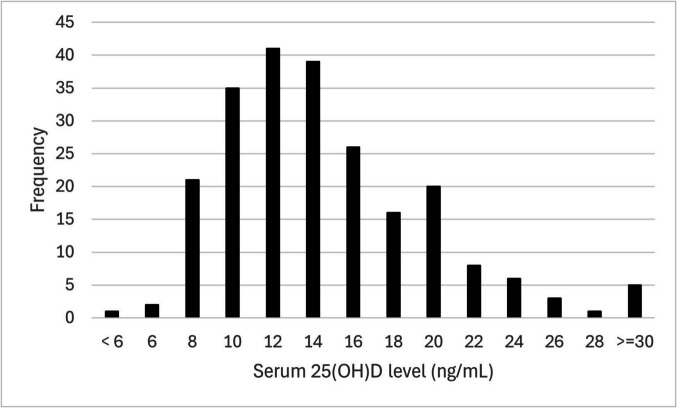
Histogram of serum 25(OH)D level. 25(OH)D: 25-hydroxyvitamin D

The demographic characteristics of the participants are presented in Table 1. The median (IQR) serum 25(OH)D concentration was 14.5 (11.8–18.3) ng/mL. Of the participants, 80.8% had vitamin D deficiency (25(OH)D < 20 ng/mL) and 26.3% had severe vitamin D deficiency (25(OH)D < 12 ng/mL). Only 2.2% of the participants had sufficient vitamin D levels (Fig. 1). When serum 25(OH)D levels were compared among the three groups (< 12, 12–20, and ≥ 20 ng/mL), a trend toward shorter outdoor activity times with lower blood levels was observed (*P* = 0.002; < 12 ng/mL group *vs*. ≥ 20 ng/mL: *P* = 0.003, 12–20 ng/mL *vs*. ≥ 20 ng/mL: *P* = 0.02). Nearly two-thirds of the respondents in all three groups habitually used sunscreen or parasols (Additional file).

**Table 1:** Demographic characteristics

n	224
Age years, median (IQR)	21 (19–22)
Living alone, n (%)	74 (33.0)
Loving with parents, n (%)	133 (59.4)
Non-breakfast eater, n (%)	70 (31.2)
Smoking, n (%)	3 (1.3)
Habitual drinking, n (%)	15 (6.7)
Regular physical exercise, n (%)	73 (32.6)
Premenstrual syndrome, n (%)	118 ( 52.7)

*IQR* interquartile range

Those with a K6 score ≥ 5 accounted for 41.1%, and those with a K6 score ≥ 13 accounted for 4.0%. The 25(OH)D level was significantly lower in the K6 score ≥ 5 group than in the K6 score < 5 group, and deficient individuals were predominant (Table 2). All patients with a K6 score of ≥ 13 were vitamin D deficient. Compared to the K6 score < 5 group, the K6 score ≥ 5 group tended to sleep less on weekdays and more than eight hours on weekends, and SJL tended to be larger. Furthermore, the AIS scores were higher in the K6 score ≥ 5 group, reflecting higher daytime sleepiness than insomnia symptoms (Table 3).

**Table 2:** Comparisons of demographic characteristics between K6 score < 5 group and K6 score ≥ 5 group

	K6 score < 5	K6 score ≥ 5	*P* value
n	132	92	
Age years, median (IQR)	21 (19, 22)	21 (19, 22)	0.70
Living alone, n (%)	51 (38.6)	23 (25.0)	0.04
Loving with parents, n (%)	72 (54.5)	61 (66.3)	0.10
Non-breakfast eater, n (%)	42 (31.8)	28 (30.4)	0.88
Smoking, n (%)	1 (0.8)	2 (2.2)	0.57
Habitual drinking, n (%)	10 (7.6)	5 (5.4)	0.60
Regular physical exercise, n (%)	42 (31.8)	31 (33.7)	0.77
Premenstrual syndrome, n (%)	66 (50.0)	52 (56.5)	0.35
K6 score, median (IQR)	2.0 (0.0, 3.0)	8.0 (6.0, 10.0)	< 0.001
Loneliness score, median (IQR)	3.0 (3.0, 4.0)	4.0 (3.0, 6.0)	< 0.001
> = 6, n (%)	6 (4.5)	29 (31.5)	< 0.001
25(OH)D ng/mL, median (IQR)	15.1 (12.6, 19.7)	13.3 (11.2, 17.7)	0.02
< 12 ng/mL, n (%)	28 (21.2)	31 (33.7)	0.02
12–20 ng/mL, n (%)	72 (54.5)	50 (54.3)	
≥ 20 ng/mL, n (%)	32 (24.2)	11 (12.0)	
Intact PTH pg/mL, median (IQR)	35.5 (28.8, 44.3)	38.0 (29.8, 47.5)	0.45
Ca mg/dL, median (IQR)	9.40 (9.20, 9.60)	9.50 (9.30, 9.70)	0.05
P mg/dL, median (IQR)	3.65 (3.40, 3.92)	3.65 (3.30, 4.10)	0.78
ferritin ng/mL, median (IQR)	19.5 (11.4, 36.3)	19.6 (11.6, 31.6)	0.89

*P* values were calculated using the Mann–Whitney U test and Fisher’s exact probability test

*IQR* interquartile range, *25(OH)D* 25-hydroxyvitamin D, *PTH* parathyroid hormone

**Table 3:** Comparisons of sleep characteristics between K6 score < 5 group and K6 score ≥ 5 group

	K6 score < 5	K6 score ≥ 5	*P* value
n	132	92	
Average Sleep duration hr, median (IQR)	6.95 (6.40, 7.57)	6.95 (6.25, 7.67)	0.70
< 6 h, n (%)	14 (10.7)	17 (18.9)	0.21
6–7 h, n (%)	57 (43.5)	29 (32.2)	
7–8 h, n (%)	44 (33.6)	31 (34.4)	
> = 8 h, n (%)	16 (12.2)	13 (14.4)	
Corrected MST, median (IQR), h:mm	4:07 (3:28, 4:52)	3:58 (3:11,5:01)	0.79
Chronotype			
Morning, n (%)	41 (31.3)	33 (36.7)	0.40
Intermediate, n (%)	48 (36.6)	25 (27.8)	
Evening, n (%)	42 (32.1)	32 (35.6)	
SJL min, median (IQR)	47.5 (23.8, 90.0)	61.5 (30.0, 114.4)	0.03
< 1 h, n(%)	77 (58.8)	39 (43.3)	0.009
1–2 h, n (%)	41 (31.3)	29 (32.2)	
> = 2 h, n (%)	13 (9.9)	22 (24.4)	
Weekday			
Sleep duration hr, median (IQR)	6.25 (5.67, 6.92)	6.00 (5.12, 6.67)	0.03
< 5 h, n (%)	15 (11.5)	20 (22.2)	0.20
5 h, n (%)	37 (28.2)	22 (24.4)	
6 h, n (%)	57 (43.5)	36 (40.0)	
> = 7 h, n (%)	22 (16.8)	12 (13.3)	
Weekend			
Sleep duration hr, median (IQR)	8.50 (7.50, 9.17)	8.52 (8.00, 9.50)	0.16
< 7 h, n (%)	15 (11.5)	11 (12.2)	0.02
7 h, n (%)	40 (30.5)	15 (16.7)	
8 h, n (%)	23 (17.6)	30 (33.3)	
> = 9 h, n (%)	53 (40.5)	34 (37.8)	
AIS score, median (IQR)	3.00 (1.00, 5.00)	5.00 (3.00, 7.00)	< 0.001
> = 6, n (%)	19 (14.4)	43 (46.7)	< 0.001
Difficulty initiating sleep, n (%)	50 (37.9)	44 (47.8)	0.17
Difficulty maintaining sleep, n (%)	6 (4.5)	12 (13.0)	0.03
Moderate to severe EDS, n (%)	22 (16.7)	39 (42.4)	< 0.001
RLS, n (%)	4 (3.0)	4 (4.3)	0.72

*P* values were calculated using the Mann–Whitney U test and Fisher’s exact probability test

*AIS* Athens Insomnia Scale, *EDS* excessive daytime sleepiness, *IQR* interquartile range, *MST* mid-sleep time, *RLS* restless legs syndrome, *SJL* social jetlag

**Fig. 2:**
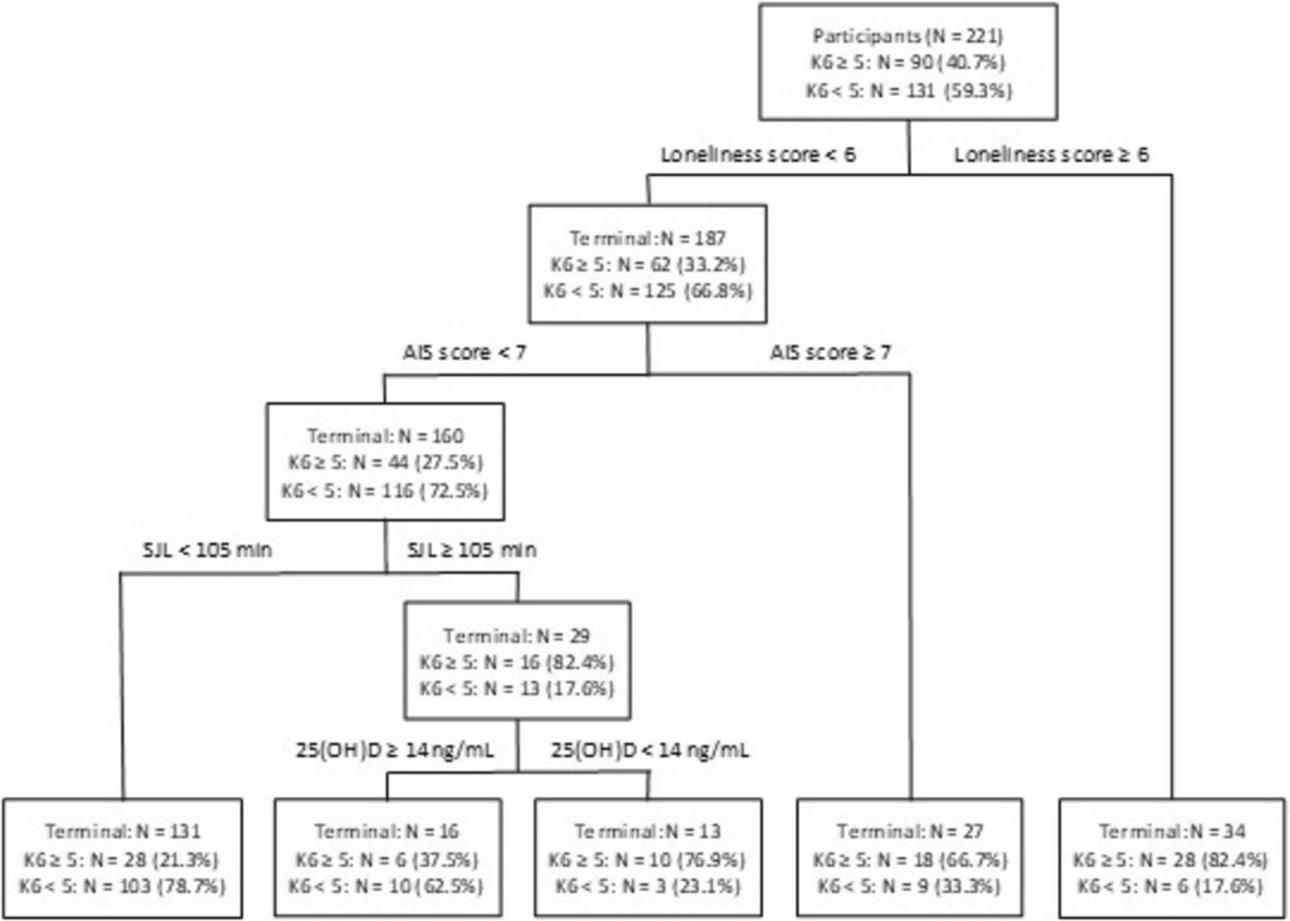
Classification and regression trees for predicting factors associated with K6 ≥ 5. AIS, Athens insomnia scale; 25(OH)D, 25-hydroxyvitamin D; SJL, social jetlag

The loneliness score, AIS score, SJL, weekday sleep duration, and blood 25(OH)D concentration, which were significant in the comparison between the two groups with K6 ≥ 5 and K6 < 5, were entered in the CART analysis. Since the Fisher’s exact probability test showed that the four categories of sleep duration on weekends were significant, we also included sleep duration on weekends. Consequently, neither weekday nor weekend sleep duration was included in the algorithm. Four nodes were created in order of mathematically significant variables to classify participants with K6 ≥ 5 and K6 < 5, namely loneliness score ≥ 6, AIS score ≥ 7, SJL ≥ 105 min, and serum 25(OH)D concentration < 14.2 ng/mL, and five terminal nodes were created (Fig. 2). The target accuracy (95% CI) of the algorithm was 76.5 (70.3–81.9)%, and the sensitivity and specificity (95% CI) were 62.2 (51.4–72.2)% and 86.3 (79.2–91.6)%, respectively.

## Discussion

This study reconfirms that vitamin D deficiency is significantly high among Japanese female university students. Although vitamin D deficiency was not significantly associated with sleep–wake parameters, it was associated with mental health problems. When participants with mental health problems were evaluated for the importance of variables using CART analysis, a form of machine learning, loneliness, insomnia symptoms, SJL, and vitamin D deficiency were employed in this order. The cutoff points for the variables employed in the CART analysis were within the range generally considered problematic for each variable. The effectiveness of interventions for each variable or multiple variables needs to be verified as a mental health measure for this age group.

The prevalence of vitamin D deficiency in our study was similar to that previously reported [7, 8]. In addition, a trend toward higher levels with longer outdoor activity times was shown. The amount of UV irradiation of sunlight required for vitamin D synthesis was reported lower than the level that causes skin damage, resulting in erythema; therefore, attempts have been made to use UV irradiation of sunlight that does not cause erythema [57, 58]. Most of the vitamin D in the body is derived from vitamin D synthesized in the skin by ultraviolet light, and the appropriate use of ultraviolet light is important to prevent vitamin D deficiency.

Although vitamin D deficiency is associated with mental health problems, the cutoff values associated with serum 25(OH)D concentrations and mental health should be carefully established. In this study, vitamin D deficiency was also common among participants with K6 < 5. In addition, in a study examining the association between depressive symptoms and vitamin D deficiency in adults aged 40 years and older, the odds ratio for depressive symptoms was significantly higher in women in the lowest quartile of 25(OH)D blood levels compared to the highest quartile; however, this association was not found in those younger than 62 years [59]. When the CART analysis was performed in the present study, a serum 25(OH)D concentration < 14 ng/mL, which is lower than the common indicator of vitamin D deficiency, < 20 ng/mL, was employed in relation to mental health. Similarly, serum 25(OH)D concentrations < 10–13 ng/mL were employed in association with RLS in pregnant women, although the study did not examine the association with depressive symptoms [8]. Vitamin D levels affecting the central nervous system may be lower than the cutoff values for vitamin D deficiency, which have been formulated based on bone metabolism.

In the present study, as in previous reports [60, 61], SJL expansion was associated with mental health problems. The SJL is generally magnified by an extreme evening chronotype and accumulated sleep debt [62]. Although no association between mental health problems and chronotype assessed by MST was found in the present participants, mental health problems were related to a tendency for shorter sleep duration on weekdays and moderate to severe daytime sleepiness. A study of United Kingdom university students found that mental health problems were associated with evening chronotype rather than SJL [63], but this study did not examine sleep duration or sleep debt. Japanese people are known to be the shortest sleepers in the world [64], and the effects of weekday sleep loss in Japanese female university students may be related to mental health problems associated with increased SJL.

The data collection period coincided with the COVID-19 pandemic, and changes in behavioral patterns during this period may have influenced our results. In the CART analysis, loneliness was the most important factor in selecting participants with mental health problems. Loneliness was reported to have increased during the pandemic [36], and increased loneliness during this period may have contributed to the present results. In addition, low serum 25(OH)D levels have been reported during this period [65], and the decreased opportunities to go outside due to behavioral restrictions may have influenced the increase in vitamin D deficient individuals by decreasing their exposure to sunlight. Furthermore, a trend toward longer and more delayed sleep phase during this period has been reported [66], and the effects of weekday sleep duration and SJL may have been underestimated. Further investigation is needed to determine whether similar results can be obtained with the same reproducibility.

This study has four limitations. First, in the present study, all parameters related to sleep were confirmed using questionnaires and were not objective indices. Although vitamin D deficiency has been reported to be associated with various sleep–wake problems [33], we did not find any association between vitamin D deficiency and sleep–wake problems. It is necessary to examine this relationship by fully differentiating sleep–wake disorders using more objective sleep parameters. Secondly, this study did not identify any indicators of body size. Vitamin D deficiency is also associated with obesity. Therefore, BMI should have been considered in the present study. Third, although the study period coincided with the spread of COVID-19, its effects were not examined. Long COVID, a sequela of COVID-19, includes many mental health and insomnia problems that may have influenced our results. Fourth, the number of participants in this study was notably reduced during the summer and winter seasons, thereby affecting the understanding of seasonal variations in vitamin D levels. Vitamin D is predominantly synthesized in the skin through exposure to ultraviolet rays, with blood concentrations typically peaking in summer when ultraviolet exposure is highest. To elucidate the impact of seasonal fluctuations, conducting a longitudinal study involving the same participants throughout the entire year would be necessary.

## Conclusions

Vitamin D deficiency is highly prevalent among female Japanese university students and is associated with mental health problems. Although the results of the present study alone do not allow us to determine the cutoff value of serum 25(OH)D concentration affecting mental health problems, the level indicating vitamin D deficiency linked to mental health may be below the widely used 25(OH)D < 20 ng/mL. Mental health problems in this age group are associated with loneliness, insomnia symptoms, SJL, and vitamin D deficiency. A comprehensive strategy is crucial for implementing mental health interventions for Japanese female university students. First, an appropriate social support system tailored to each individual is required. Additionally, sleep hygiene instructions and cognitive-behavioral therapy approaches, which also contribute to SJL reduction, may be effective. Furthermore, to eliminate vitamin D deficiency, it is necessary to provide information on safe sun exposure that does not cause skin erythema and increase the intake of vitamin D-containing foods in the diet.

## Supplementary Information

Additional file 1.

## Data Availability

No datasets were generated or analysed during the current study.

## Abbreviations

AIS: Athens Insomnia Scale
CI: Confidence interval
EDS: Excessive daytime sleepiness
IQR: Interquartile range
LC–MS/MS: Liquid chromatography-tandem mass spectrometry
MST: Mid-sleep time
25(OH)D: 25-Hydroxyvitamin D
PMS: Premenstrual syndrome
PTH: Parathyroid hormone
RLS: Restless legs syndrome
SJL: Social jetlag
