# Supplementary material for: Loneliness, insomnia symptoms, social jetlag, and vitamin D deficiency in relation to mental health problems in Japanese female university students: a cross-sectional study
Source: J Physiol Anthropol. 2025 Jul 9;44:19. doi: 10.1186/s40101-025-00403-9 (PMC12239299; doi:10.1186/s40101-025-00403-9)
Supplement: Supplementary file 1 — Additional file 1. [file 40101_2025_403_MOESM1_ESM.docx]

**Additional file**

**Loneliness, insomnia symptoms, social jetlag, and vitamin D deficiency in relation to mental health problems in Japanese female university students: a cross-sectional study**

Additional file . Comparisons of demographic and sleep characteristics between three groups of serum 25(OH)D concentration

|  | < 12 ng/mL | 12-20 ng/mL | >= 20 ng/mL | *P* value | Multiple comparisons, *P* value |
| --- | --- | --- | --- | --- | --- |
| n | 59 | 122 | 43 |  |  |
| Age years, median (IQR) | 21 (20, 22) | 20 (19, 22) | 21 (19, 23) | 0.52 |  |
| Living alone, n (%) | 23 (39.0) | 35 (28.7) | 16 (37.2) | 0.30 |  |
| Living with parents, n (%) | 33 (55.9) | 77 (63.1) | 23 (53.5) | 0.42 |  |
| Non-breakfast eater, n (%) | 22 (37.3) | 37 (30.3) | 11 (25.6) | 0.45 |  |
| Smoking, n (%) | 1 (1.7) | 2 (1.6) | 0 (0.0) | 1 |  |
| Habitual drinking, n (%) | 5 (8.5) | 8 (6.6) | 2 (4.7) | 0.82 |  |
| Regular physical exercise, n (%) | 9 (15.3) | 41 (33.6) | 23 (53.5) | < 0.001 |  |
| Weekly outdoor activity time, hr (IQR) | 3.50 (2.50, 7.50) | 5.00 (3.10, 8.69) | 7.00 (4.83, 11.25) | 0.002 | < 12 *vs*. ≥ 20: 0.003, 12–20 vs. *vs*. ≥ 20 ng/mL: 0.02 |
| Use of sunscreen or parasol, n (%) | 37 (62.7) | 79 (64.8) | 27 (62.8) | 0.95 |  |
| PMS, n (%) | 32 (54.2) | 64 (52.5) | 22 (51.2) | 0.97 |  |
| K6 score, median (IQR) | 5.0 (2.0, 7.0) | 3.0 (1.0, 6.0) | 3.0 (0.5, 4.5) | 0.17 |  |
| >= 5, n (%) | 31 (52.5) | 50 (41.0) | 11 (25.6) | 0.02 |  |
| Loneliness score, median (IQR) | 4.0 (3.0, 5.0) | 3.0 (3.0, 5.0) | 4.0 (3.0, 4.5) | 0.69 |  |
| >= 6, n (%) | 12 (20.3) | 17 (13.9) | 6 (14.0) | 0.53 |  |
| 25(OH)D ng/mL, median (IQR) | 10.2 (9.4, 11.2) | 14.9 (13.7, 16.5) | 22.3 (21.8, 25.1) | < 0.001 |  |
| Intact PTH pg/mL, median (IQR) | 40.0 (31.0, 50.0) | 35.0 (27.0, 44.8) | 34.0 (29.0, 39.0) | 0.02 | < 12 *vs*. 12–20 ng/mL; 0.03 |
| Ca mg/dL, median (IQR) | 9.4 (9.2, 9.6) | 9.5 (9.3, 9.7) | 9.4 (9.2, 9.7) | 0.02 | < 12 *vs*. 12–20 ng/mL; 0.02 |
| P mg/dL, median (IQR) | 3.5 (3.2, 3.9) | 3.7 (3.4, 4.0) | 3.7 (3.5, 3.9) | 0.19 |  |
| ferritin ng/mL, median (IQR) | 17.5 (10.6, 28.0) | 20.1 (11.8, 37.1) | 24.0 (11.9, 37.5) | 0.34 |  |
| Average sleep duration hr, median (IQR) | 7.17 (6.24, 7.59) | 6.96 (6.42, 7.64) | 6.72 (6.17, 7.51) | 0.44 |  |
| < 6 hr, n (%) | 10 (17.2) | 13 (10.7) | 8 (19.0) | 0.24 |  |
| 6-7 hr, n (%) | 16 (27.6) | 51 (42.1) | 19 (45.2) |  |  |
| 7-8 hr, n (%) | 25 (43.1) | 39 (32.2) | 11 (26.2) |  |  |
| >= 8 hr, n (%) | 7 (12.1) | 18 (14.9) | 4 (9.5) |  |  |
| Corrected MST, median (IQR), h:mm | 4:11 (3:18, 5:00) | 4:00 (3:22,4:40) | 4:12 (3:27, 5:00) | 0.77 |  |
| Chronotype |  |  |  |  |  |
| Morning, n (%) | 19 (32.8) | 40 (33.1) | 15 (35.7) | 0.15 |  |
| Intermediate, n (%) | 15 (25.9) | 48 (39.7) | 10 (23.8) |  |  |
| Evening, n (%) | 24 (41.4) | 33 (27.3) | 17 (40.5) |  |  |
| SJL min, median (IQR) | 62.5 (30.0, 91.9) | 57.5 (30.0, 90.0) | 45.0 (15.0, 88.8) | 0.28 |  |
| < 1 hr, n(%) | 26 (44.8) | 63 (52.1) | 27 (64.3) | 0.46 |  |
| 1-2 hr, n (%) | 21 (36.2) | 39 (32.2) | 10 (23.8) |  |  |
| >= 2 hr, n (%) | 11 (19.0) | 19 (15.7) | 5 (11.9) |  |  |
| Weekday |  |  |  |  |  |
| Sleep duration hr, median (IQR) | 6.00 (5.33, 6.67) | 6.25 (5.50, 6.83) | 6.00 (5.25, 7.00) | 0.44 |  |
| < 5 hr, n(%) | 12 (20.7) | 16 (13.2) | 7 (16.7) | 0.12 |  |
| 5 hr, n(%) | 16 (27.6) | 30 (24.8) | 13 (31.0) |  |  |
| 6 hr, n (%) | 22 (37.9) | 60 (49.6) | 11 (26.2) |  |  |
| >= 7 hr, n (%) | 8 (13.8) | 15 (12.4) | 11 (26.2) |  |  |
|  |  |  |  |  |  |
| Weekend |  |  |  |  |  |
| Sleep duration hr, median (IQR) | 8.67 (8.00, 9.50) | 8.50 (7.50, 9.50) | 8.08 (7.50, 9.00) | 0.15 |  |
| < 7 hr, n (%) | 5 (8.6) | 14 (11.6) | 7 (16.7) | 0.49 |  |
| 7 hr, n (%) | 10 (17.2) | 32 (26.4) | 13 (31.0) |  |  |
| 8 hr, n (%) | 16 (27.6) | 29 (24.0) | 8 (19.0) |  |  |
| >= 9 hr, n (%) | 27 (46.6) | 46 (38.0) | 14 (33.3) |  |  |
| AIS score, median (IQR) |  |  |  |  |  |
| >= 6, n (%) | 17 (28.8) | 32 (26.2) | 13 (30.2) | 0.85 |  |
| Difficulty initiating sleep, n (%) | 53 (89.8) | 113 (92.6) | 41 (95.3) | 0.58 |  |
| Difficulty maintaining sleep, n (%) | 59 (100.0) | 120 (98.4) | 43 (100.0) | 1 |  |
| Moderate to severe EDS, n (%) | 14 (23.7) | 36 (29.5) | 11 (25.6) | 0.71 |  |
| RLS, n (%) | 2 (3.4) | 2 (1.6) | 4 (9.3) | 0.06 |  |

*P* values were calculated using the Kruskal-Wallis test and Fisher's exact probability test. The Bonferroni correction was used for multiple comparisons. AIS: Athens Insomnia Scale, EDS: excessive daytime sleepiness, IQR: interquartile range, MST: mid-sleep time, 25(OH)D: 25-hydroxyvitamin D, PMS: premenstrual syndrome, PTH: parathyroid hormone, RLS: restless legs syndrome, SJL: social jetlag.
